# Genome-Scale Metabolic Modeling Reveals Sequential Dysregulation of Glutathione Metabolism in Livers from Patients with Alcoholic Hepatitis

**DOI:** 10.3390/metabo12121157

**Published:** 2022-11-22

**Authors:** Alexandra Manchel, Radhakrishnan Mahadevan, Ramon Bataller, Jan B. Hoek, Rajanikanth Vadigepalli

**Affiliations:** 1 Daniel Baugh Institute for Functional Genomics and Computational Biology, Department of Pathology, Anatomy, and Cell Biology, Thomas Jefferson University, Philadelphia, PA 19107, USA; 2 Department of Chemical Engineering and Applied Chemistry, University of Toronto, Toronto, ON M5S 3E5, Canada; 3 The Institute of Biomedical Engineering, University of Toronto, Toronto, ON M5S 3G9, Canada; 4 The Liver Unit, Hospital Clinic, 08036 Barcelona, Spain

**Keywords:** alcoholic hepatitis, genome-scale metabolic modeling, flux balance analysis, RNA-seq, network analysis, trajectory analysis, liver disease, metabolism, systems biology

## Abstract

Alcoholic hepatitis (AH) is the most severe form of alcoholic liver disease for which there is no efficacious treatment aiding most patients. AH manifests differently in individuals, with some patients showing debilitating symptoms more so than others. Previous studies showed significant metabolic dysregulation associated with AH. Therefore, we sought to analyze how the activity of metabolic pathways differed in the liver of patients with varying degrees of AH severity. We utilized a genome-scale metabolic modeling approach that allowed for integration of a generic human cellular metabolic model with specific RNA-seq data corresponding to healthy and multiple liver disease states to predict the metabolic fluxes within each disease state. Additionally, we performed a systems-level analysis of the transcriptomic data and predicted metabolic flux data to identify the regulatory and functional differences in liver metabolism with increasing severity of AH. Our results provide unique insights into the sequential dysregulation of the solute transport mechanisms underlying the glutathione metabolic pathway with increasing AH disease severity. We propose targeting of the solute transporters in the glutathione pathway to mimic the flux activity of the healthy liver state as a potential therapeutic intervention for AH.

## 1. Introduction

The diagnosis and treatment of alcohol liver disease (ALD), more specifically alcoholic hepatitis (AH), remain severe clinical issues as very few efficacious therapeutics are available. Patients with AH may experience minimal symptoms, which further complicates the diagnosis and treatment of the disease [1]. Current therapeutic regimens focus on adherence to a strict diet plan and abstinence from alcohol [2]. While some studies show that supplementation with corticosteroids may reduce inflammation in patients with severe AH, many patients are non-responsive to such treatments or the treatments lack long-term efficacy [3]. If the patient does not strictly follow the standard-of-care treatment plan, diagnosis may occur too late and the disease may progress to an irreversible stage. If the patient is non-responsive to corticosteroid supplementation, liver transplantation may be the only option for full functional rehabilitation of the organ [4]. However, the number of adults needing liver transplants has increased (by roughly 63% from 2007 to 2017 in the United States (US)) even as the number of donors has stayed the same [5]. ALD has now become the most common indication for liver transplantation in the US, replacing the hepatitis C virus (HCV) [6]. Therefore, it is essential to elucidate the pathological pathways and mechanisms driving the progression of ALD such that therapeutics can be engineered to halt disease progression or, in the best case, revert the diseased liver to a healthy, homeostatic state, thereby eliminating the need for transplantation.

The progression of ALD follows a distinct trajectory; following continued alcohol consumption in addition to fat accumulation in the hepatocytes, one’s liver may progress to a state of alcoholic steatohepatitis (ASH). Alcoholic liver disease can then progress to AH, which affects roughly 10 to 35% of heavy drinkers [7]. AH is marked by significant immune cell infiltration, activation, and inflammation of hepatocytes. AH may progress to alcoholic cirrhosis (AC), which is an irreversible stage of alcoholic liver disease in which there is scarring of the liver.

It is essential to distinguish the sequential progression of AH from non-alcoholic liver disease (NALD) states. While AH and NALD are both characterized as a form of liver disease, the etiology and progression of the diseases are distinct [8,9,10,11,12]. Furthermore, studies suggest that AH and NALD have differentiating signaling pathways leading to disease onset [13]. Therefore, it is essential to compare and contrast NALD with AH states at both the regulatory and functional level.

AH is distinct from NALD in that it is characterized by a cascade of pathological events associated with the metabolism of ethanol and acetaldehyde, leading to the production of reactive oxygen species (ROS) and the generation of lipids [14]. Conversely in NALD, lipid accumulation is mostly due to excess nutritional intake and/or complications due to insulin resistance causing changes in systemic lipid metabolism [15]. Following continuous ethanol exposure and metabolic dysregulation in processes leading to downstream endoplasmic reticulum stress, lipid peroxidation, and DNA damage, acute liver failure in the form of AH may arise [16].

A previous study showed that glucose metabolism is tightly regulated in healthy hepatocytes, the parenchymal cells of the liver. However, following the onset of AH, intermediates in the glycolysis/gluconeogenesis pathway, tricarboxylic acid cycle, and mono/disaccharide pathway become severely dysregulated. Specifically, hexokinase domain containing 1 (HKDC1), which converts glucose into glucose-6-phosphate (G6P) in the glycolysis pathway, was identified as the most up-regulated kinase in patients with AH [17]. An additional study found that the use of the hepatocyte HNF4a P2 promoter led to defective metabolic and synthetic functions in AH patients [18]. These studies prompted our further examination of metabolic dysregulation in AH. Specifically, we sought to characterize metabolic pathway activity across varying degrees of AH to determine which metabolic subsystems are sequentially dysregulated with the disease progression and severity.

In order to study AH at the metabolic level, we utilize genome-scale metabolic modeling and flux balance analysis methodologies. Previous studies showed that genome-scale metabolic models (GEMs) provide a valuable framework for integrative analysis and understanding of underlying metabolic mechanisms in the liver [19,20,21,22,23]. While GEMs have been developed to model the healthy liver, healthy hepatocyte, and non-alcoholic liver disease states, this is the first study of its kind to generate GEMs specific to AH. A previously published study on NALD GEMs revealed that diseased patients have a reduced metabolic adaptability, i.e., the fat accumulated during the disease increases the demand of the liver to regulate metabolic responses to maintain normal liver function [24]. The accumulation of hepatic fat induced mitochondrial metabolism, lipolysis, glyceroneogenesis, and a switch from lactate to glycerol as a substrate for gluconeogenesis. Another study showed that there is significant dysregulation of metabolites within the glycosphingolipid pathway during NALD progression [25]. Specifically, there were alterations in vitamins A and E and glycosphingolipid signatures in the liver and blood of patients with advanced non-alcoholic fibrosis.

We aim to examine the changes in hepatic metabolism across progressing AH disease states. In doing so, we will explore locus points of metabolic dysregulation as disease severity increases, allowing for the possibility of therapeutic intervention specific to the disease state. Furthermore, we seek to discover which metabolic activity is unique to progressive AH but not NALD, and if there are novel metabolic subsystems or biological pathways that sequentially increase flux with AH progression in order to compensate for the metabolic dysregulation.

## 2. Materials and Methods

### 2.1. The Patient Samples and RNA-Seq Data

A total of 90 human liver biopsy samples were obtained as per the “Human Biorepository Core from the NIH-funded international InTeam consortium” (7U01AA021908-05). AH patients were chosen for the study based on strict inclusion and exclusion criteria. Inclusion criteria for AH patients were: (1) between the ages of 18 and 70, (2) active alcohol abuse in the past 3 months (>50 g/day for men and >40 g/day for women), (3) Aspartate Aminotransferase (AST) > Alanine Aminotransferase (ALT), (4) total bilirubin level > 3.0 mg/dl in the past 3 months, and (4) liver biopsy and/or a clinical picture consistent with alcohol hepatitis. Exclusion criteria were: (1) autoimmune liver disease (antinuclear antibody (ANA) > 1:320), (2) hepatitis B infection, (3) hepatocellular carcinoma, (4) complete portal vein thrombosis, (5) advanced or terminal extrahepatic diseases, (6) lack of consent to participate in the study, (7) pregnancy, and (8) receiving more than 3 days of treatment with prednisolone or pentoxifyllin prior to study start date. In all patients, the clinical picture was consistent with AH, and in patients who underwent liver biopsy, the histology was in line with the diagnosis of AH. Liver biopsies were only done if clinically indicated as part of routine clinical care for diagnostic purposes of AH. Serum samples were also collected to test for liver function. Model for End-stage Liver Disease (MELD), Child–Pugh, and ABIC scores were calculated for all patients where the required variables were available. The protocol was approved by the Ethics Committee of each participating center from the InTeam consortium and by the central Institutional Review Board of the University of North Carolina at Chapel Hill, and written informed consent was obtained from each patient.

RNA sequencing was performed according to [18]. Specially, as stated, total RNA from flash-frozen liver tissue was extracted by phenol/chloroform separation (TRIzol, Thermo Fisher Scientific Inc., Waltham, MA, USA). RNA purity and quality were assessed by automated electrophoresis (Bioanalyzer, Agilent Technologies Inc., Santa Clara, CA, USA) and were sequenced using the Illumina HiSeq2000 platform. Libraries were built using TruSeq Stranded Total RNA Ribo-Zero GOLD (Illumina Inc., San Diego, CA, USA). Sequencing was paired end (2 × 100 bp) and multiplexed. Ninety-four paired-end sequenced samples obtained an average of 36.9 million total reads, with 32.5 million (88%) mapped to GRCh37/hg19 human reference. Short read alignment was performed using the STAR alignment algorithm with default parameters [26]. To quantify expression from transcriptome mappings, we employed the software package RNA-seq by Expectation Maximization (RSEM) [27].

The 90 patient samples were stratified into 7 distinct disease states and a healthy control state: explant alcoholic hepatitis (explant AH, *n* = 10), severe alcoholic hepatitis (severe AH, *n* = 18), non-severe alcoholic hepatitis (non-severe AH, *n* = 11), early alcoholic steatohepatitis (early ASH, *n* = 12), healthy (*n* = 10), compensated cirrhosis (comp. cirrhosis, *n* = 9), hepatitis C virus (HCV, *n* = 10), and non-alcoholic steatohepatitis (NASH, *n* = 9). Patients had a median age of 45 in the explant AH disease state category, median age of 51 in severe AH, median age of 46 in non-severe AH, median age of 52 in early ASH, median age of 32 in the healthy controls, median age of 61 in compensated cirrhosis, median age of 46 in HCV, and median age of 49 in NASH. Samples classified as explant AH originated from patients undergoing a liver transplant. At the time of transplant, the liver was removed from the body and was identified as having AH. A biopsy sample was then taken from the explanted liver. Samples exhibiting severe AH were clinically and pathology proven AH and had a MELD score > 21. The severe AH patient cohort contained both responders and non-responders to corticosteroids. The non-severe AH patients were heterogeneous in that some displayed AH criteria (criteria discussed above) while some did not, but all had a MELD score < 21. The early ASH patients were non-obese, actively abused alcohol, and displayed liver damage (elevation of liver enzymes) but did not have liver dysfunction (i.e., asymptomatic, normal bilirubin, normal prothrombin levels). They also presented mild elevation of transaminases and histologic criteria of ASH but never had AH episodes. The control samples were taken before any clamping or ischemia from the normal liver surrounding apparent liver nodules as a result of metastases. The patient samples from the compensated cirrhosis, HCV, and NASH cohorts were all clinically and pathology proven and none of the patients had any AH episodes. Patients from the compensated cirrhosis cohort were asymptomatic and had HCV and cirrhosis, but did not have ascites, variceal hemorrhage, hepatic encephalopathy, or jaundice. Patients within the HCV cohort did not have cirrhosis, and patients within the NASH cohort were defined according to Keiner’s Criteria and displayed accumulated hepatic fat histologically and had above average body mass index (BMI > 25).

For additional information about the patient samples, we refer the reader to the clinical trial website (https://clinicaltrials.gov/ct2/show/NCT02075918 (accessed on 19 November 2022)).

### 2.2. Generating Genome-Scale Metabolic Models

The mapped RNA-seq raw counts file was transcript-per-million (TPM) normalized and integrated with the generic Human1 metabolic model using the RAVEN toolbox in MATLAB [28,29]. Specifically, the Human1 model and RNA-seq data were loaded into MATLAB, a threshold of 1 TPM was set, and the *tINIT* algorithm was run to extract GEMs specific to each liver disease state based on the corresponding RNA expression profile [20]. Briefly, the *tINIT* algorithm works as follows: each reaction in the metabolic model has an associated gene rule, which contains one or more genes separated by AND or OR. If the gene rule only contains a single gene, the maximum TPM value must be greater than 1 for the associated reaction to be included in the model. If the gene rule contains multiple genes separated by AND, representing a complex enzyme, then the minimum of the maximum gene expression values must be greater than 1 for the reaction to be included in the model. If the gene rule contains multiple genes separated by OR, representing isozymes, then the maximum of the maximum gene expression values must be greater than 1 for the reaction to be included in the model. Following integration of the RNA-seq data with the Human1 model, a metabolic task list was provided such that the extracted model was able to perform all of the necessary tasks of a viable cell. If the task was unable to be performed, the necessary metabolites, genes, and reactions were included in the model, despite previously not being included due to the set TPM threshold. The metabolic task list is provided in Supplementary File S1. Finally, the *checkTasks* function was employed on all GEMs to confirm that all metabolic tasks were able to be performed.

The structure of each GEM—i.e., the number of genes, metabolites, and reactions—were compared to one another using the *compareMultipleModels* function from the RAVEN toolbox. The output is a binary vector (consisting of only 0′s and 1′s) and which reactions are included in the models; missing reactions are given a 0 and present reactions are given a 1. This enabled the use of quantitative metrics, such as Hamming similarity, to compare reaction coverage across the generated models.

A linear optimization solver is required for GEM extraction by *tINIT*. We used the Gurobi Optimizer, but MOSEK or GLPK can also be used. Additionally, we performed all GEM computation on a 56-core server with an Intel Xeon CPU E5-2697 to minimize the amount of time needed to generate our models. The MATLAB script provided in Supplementary File S2 generates all GEMs using the *tINIT* algorithm. The MATLAB script provided in Supplementary File S3 calculates the structural comparisons between all GEMs using the *compareMultipleModels* function.

### 2.3. Calculating the Objective Function and Performing Flux Balance Analysis

In order to calculate the metabolic fluxes using the generated GEMs, we must provide an objective function that will be either maximized or minimized. While other cancer-related GEM studies utilize a biomass function that maximizes cell growth, the function for which our models are to be optimized is more complex [30]. Since calculating fluxes for the GEMs is more complicated than simply assigning a single reaction to be optimized, we utilized the *SPOT* (Simplified Pearson cOrrelation with Transcriptomic data) algorithm [31]. Briefly, the algorithm calculates a suitable objective function by maximizing the Pearson correlation between a flux vector and its corresponding gene expression data. Next, we used *E-Flux2* (the E-Flux method combined with minimization of l2 norm), which performs standard flux balance analysis (FBA) and then minimizes the Euclidean norm of the flux vector to find a unique solution [31]. More explicitly, the objective function was calculated according to Equation (1) and flux balance analysis was performed according to Equations (2)–(5).

(1) $$f^{\prime }=\max\left(\frac{v\cdot g}{||v||||g||}\right)$$

(2) $$z^{*}=\max\left(f^{\prime }\cdot v\right)$$

(3) S·v=0

(4) $$g_{\min}\leq v_{j}\leq g_{\max}$$

(5) $$\min\;(\sum_{j=1}^{n}v_{j}^{2})$$

In Equation (1), the objective function is calculated by maximizing the Pearson correlation between a flux vector and its gene expression. FBA is performed by first maximizing the product of the objective function and the flux vector Equation (2), then solving for the flux vector (*v*), provided that the product of the stoichiometric matrix (S) and flux vector (*v*) is at steady state or equal to zero Equation (3) and the fluxes are bounded by the minimum and maximum gene expression data Equation (4). Lastly, the Euclidean (l2) norm is minimized to further confine the solution space of the flux vector as in Equation (5).

MATLAB was used to calculate the objective function by the *SPOT* algorithm and FBA by the *E-Flux2* algorithm. Supplementary File S4 includes the MATLAB code for calculating the model objective functions and performing FBA.

Supplementary File S5 contains a zipped file with all MATLAB functions and scripts required for running the code in Supplementary Files S2–S4.

### 2.4. Functional Annotation

The DAVID and PANTHER functional annotation tools were utilized to identify significant biological processes with false discovery rates (FDR) less than 0.05 given a gene list as input [32,33].

### 2.5. Protein–Protein Interaction Network Analysis

Protein–protein interaction (PPI) networks were identified and visualized using the STRING application with default parameters in Cytoscape [34,35,36]. Each node in the network represents a protein and edges indicate functional and physical protein associations. We required a network interaction score of 0.4 or greater (medium confidence). The confidence score is defined by the approximate probability that a predicted link exists between two enzymes in the same metabolic map in the KEGG database. The Molecular Complex Detection (MCODE) algorithm was applied to STRING networks using default parameters to identify the highest connected network component [37].

### 2.6. Dimensionality Reduction and Trajectory Analysis

Principal component analysis (PCA) dimensionality reduction was performed using the *pcaMethods* package. Pseudo-temporal trajectory analyses using minimum spanning trees (MST) were performed using the *ica* and *mst* RStudio packages. The MSTs were plotted using axes of a non-symmetric correspondence analysis (nsca). The nsca axes provide a way of plotting the MST, where each observation (or disease state) is represented at the centroid of its neighbors such that the observations are spaced out as much as possible in order to show the links between them.

### 2.7. Statistical Testing

Pairwise Wilcoxon rank sum tests were performed using the *stats* package in RStudio to test for statistical significance (*p* < 0.05).

### 2.8. Data Manipulation and Plotting

Additional RStudio packages including *limma*, *matrixStats*, *tidyverse*, *dplyr*, and *reshape2* were utilized for data manipulation. All figures were generated using *ggplot2*, *dataVisEasy*, and *pheatmap* packages in RStudio ver. 4.2.1 (RStudio, MA, USA).

### 2.9. Data Availability and Model Reproducibility

All model results and figures presented in the current work were replicated independently by a member of our research group not involved in the original modeling and simulation study; the reproduced results were in agreement with the results presented here. All data files necessary for reproducing the generated models and predicted fluxes are available as a supplement to the present manuscript or can be downloaded from GitHub (https://github.com/Daniel-Baugh-Institute/AlcoholicHepatitis_LiverGEMStudy (accessed on 19 November 2022)). For the purpose of assessing reproducibility, necessary files were downloaded from GitHub (San Francisco, CA, USA).

Stepwise instructions for generating the GEMs and performing structural comparisons between the models are provided as a supplement to this manuscript for the purpose of model reproducibility (Supplementary File S6). Software (MATLAB (ver. R2021a, Eigenvector Inc., Wenatchee, WA, USA) and RStudio (ver. 4.2.1)) versions and RStudio package versions used are provided in Supplementary File S6 as well. All eight GEM files are present in Supplementary File S7. Additionally, all figures in the present manuscript can be replicated using the RStudio script provided in Supplementary File S8. All files necessary for figure replication are present in Supplementary File S9. Self-assessment of the Ten Simple Rules for Credible Practice in Modeling and Simulation in Healthcare was performed and is included in the supplement (Supplementary File S10) [38].

## 3. Results

### 3.1. Regulatory Analysis of Patients with AH

While AH manifests from frequent and heavy alcohol use, NALD manifests from a poor, high-fat diet or viral infection. Despite the varying disease originations and differing biological implications, treatment has remained nearly identical for patients with alcoholic and non-alcoholic liver disease. We first sought to identify specific biological pathways that differed at the transcriptomic level between patients with NALD and progressive AH to examine points of metabolic regulation. Additionally, we wanted to determine how the metabolic transcriptome of AH and NALD patients differed in their regulatory behavior as compared to healthy control samples.

**Figure 1:**
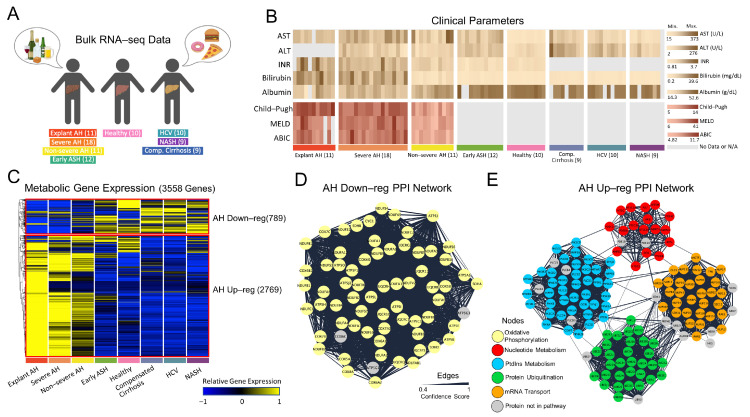
Regulatory behavior of livers from patients with AH. (**A**) Patient disease states for which RNA-seq was performed. (**B**) Clinical parameters from serum samples for each patient. (**C**) Hierarchical clustering of RNA-seq data reveals two clusters: AH Down-reg with 789 genes and AH Up-reg with 2769 genes. (**D**) Most interconnected PPI interaction network, as calculated by MCODE Cytoscape application, for AH Down-reg gene cluster. (**E**) Most interconnected PPI interaction network for AH Up-reg gene cluster. In Figure 1 (**D**,**E**), nodes (proteins) are colored by their function in a specific metabolic pathway while edge thickness is determined by the confidence score, which is computed by the STRING database.

Human liver RNA-seq samples were obtained from patients with the AH, healthy, and NALD states shown in Figure 1A. Analytical parameters of liver injury (i.e., AST and ALT), hepatocellular synthetic function (i.e., INR, serum bilirubin, and albumin), and clinical scoring systems (Child–Pugh, MELD, and ABIC) were shown for each patient (Figure 1B). All parameters were markedly impaired after AH onset.

Since many components of liver disease affect various metabolic pathways, we identified how the metabolic gene expression clustered across disease states (Figure 1C). When plotting the relative gene expression and hierarchically clustering the genes, there was distinct separation between (1) AH states (Non-severe AH, Severe AH, and Explant AH) and (2) early ASH, healthy, and NALD states, with very little overlap between the two. To simplify the nomenclature, we refer to cluster (1) as the AH Up-reg cluster and cluster (2) as the AH Down-reg cluster. The majority of metabolic genes show higher relative expression in the AH Up-reg cluster (2769 genes) as compared to the AH Down-reg cluster (789 genes). Interestingly, the healthy state exhibits similar gene expression patterns to the NALD and Early ASH state, suggesting similar mechanisms of metabolic regulation.

The genes within the AH Up-reg cluster were input into the DAVID functional annotation tool and the top five most significant (FDR < 0.05) biological processes included protein phosphorylation (GO:0006468), protein autophosphorylation (GO:0046777), peptidyl-serine phosphorylation (GO:0018105), peptidyl-tyrosine phosphorylation (GO:0018108), and intracellular signal transduction (GO:0035556), suggesting a pertinent role in protein activation. The top five most significant biological processes within the AH Down-reg cluster included mitochondrial ATP synthesis coupled proton transport (GO:0042776), aerobic respiration (GO:0009060), xenobiotic metabolic process (GO:0006805), mitochondrial electron transport, NADH to ubiquinone (GO:0006120), and steroid metabolic process (GO:0008202). A comprehensive list of all biological processes significantly enriched (FDR < 0.05) in the AH Up-reg and Down-reg clusters can be found in Supplementary File S11.

The STRING and MCODE applications in Cytoscape were utilized to visualize the PPIs and subsequently find clusters of highly interconnected regions within the networks. The subnetworks from the AH Up-reg and Down-reg clusters with the highest MCODE score (i.e., most interconnected network component) were further analyzed by functional annotation using the PANTHER database. The edge width of the network diagrams was scaled by the STRING database confidence score. Figure 1D shows the most interconnected PPI network for the AH Down-reg cluster (MCODE score = 65.582), which consists of 68 proteins (nodes) and 2197 interactions (edges). The proteins from this subnetwork were input into the PANTHER database and shown to be enriched in the oxidative phosphorylation pathway (GO:0006119). Exactly 65 of the total 68 proteins were involved in this metabolic pathway (yellow nodes). Figure 1E shows the subnetwork with the highest MCODE score (score = 34.104) for the AH Up-reg cluster. The subnetwork contains 136 proteins, 2302 interactions, and has an MCODE score of 34.104. Within this subnetwork are four distinct clusters of proteins. Functional annotation of the first cluster showed enrichment in nucleotide metabolism (GO:0009117), with 17 of the total 19 proteins belonging to the metabolic pathway (red nodes). The second cluster showed enrichment of phosphatidylinositol (PtdIns) metabolism (GO:0046488), with 41 of the total 45 proteins belonging to the metabolic pathway (blue nodes). The third cluster was enriched for protein ubiquitination (GO:0016567), with 31 of the total 33 proteins in the metabolic pathway (green nodes). The fourth and final cluster was enriched for mRNA transport (GO:0051028), with 31 of the total 39 genes belonging to the metabolic pathway (orange nodes).

The network analysis provided herein highlights dysregulated metabolic pathways that result from up- or down-regulated genes and proteins in AH. For instance, genes and proteins in the oxidative phosphorylation pathway are down-regulated with AH, which has been proven experimentally and discussed more extensively in various review articles [14,39,40]. Up-regulation of genes and proteins in the nucleotide metabolism, PtdIns metabolism, protein ubiquitination, and mRNA transport pathways were viewed in patients with AH but are vastly understudied. However, Meikle et al., 2015 reported that PtdIns was significantly associated with alcoholic liver cirrhosis, while French and Bardag-Gorce 2005, Donohue 2002, and Park et al., 2021 detailed an increase in ubiquitination associated with alcoholic liver disease [41,42,43,44].

### 3.2. Structural Analysis of Generated Metabolic Models

**Figure 2:**
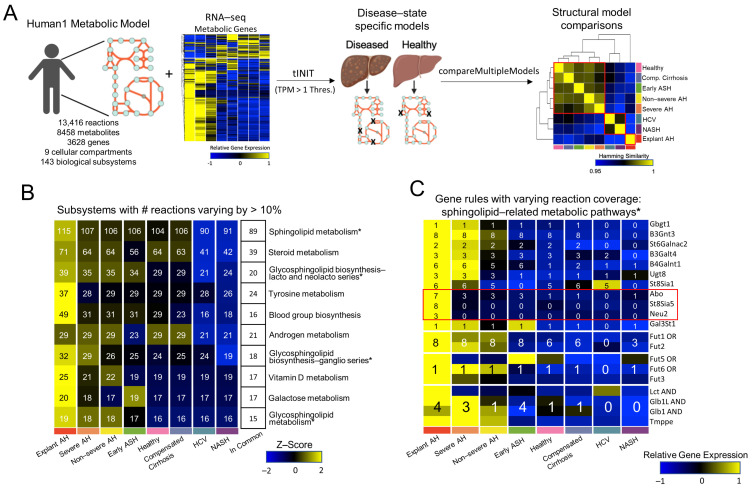
GEM generation and structural analysis. (**A**) Workflow describing the process of generating GEMs by integrating the generic Human1 metabolic model with the RNA seq data from Figure 1C by tINIT, using a TPM threshold of 1, and then making structural comparisons across models using the hamming similarity metric. (**B**) The top 10 subsystems with the number of reactions varying across any two GEMs by at least 10%. The number of reactions is shown for the top 10 subsystems for each model. The reactions in common across all models for each subsystem is shown. Subsystems are sorted by the total number of reactions within each subsystem. (**C**) The reactions that were not in commons across all models and belonged to sphingolipid-related metabolic pathways in Figure 2B were identified. The gene expressions encoding the gene rules from these reactions are shown. The number of reactions for each gene rule for each model is overlaid. * denotes a subsystem that is a sphingolipid-related metabolic pathway in Figure 2B.

The RNA-seq data was averaged across disease states and integrated with a generic genome-scale metabolic model (Human1 model) using the *tINIT* algorithm. The Human1 model, prior to integration with any data, encapsulates all reactions, metabolites, and genes that are necessary for a viable human cell that is not specific to any organ or disease state. This model contains 13,416 reactions, 8458 metabolites, 3628 genes, and 9 cellular compartments, including the cytosol, mitochondria, peroxisome, lysosome, endoplasmic reticulum, nucleus, extracellular matrix, Golgi apparatus, and inner mitochondria, as shown in Figure 2A (left). Additionally, each reaction from the Human1 model belongs to only one of the 143 biological subsystems. The transcriptomic data was integrated with the Human1 model using a transcript per million threshold (TPM) of 1 to generate a single GEM for each disease state as well as the healthy control state (Figure 2B middle). The structure of these GEMs, i.e., the number of reactions each GEM had in common, was analyzed by hamming distance, which was calculated by the *compareMultipleModels* function from the RAVEN toolbox. The results were plotted in the form of a heatmap and both the rows and columns were hierarchically clustered to identify which GEMs were most similar (Figure 2C right). While the explant AH model clustered alone (most dissimilar to all other model structures), the healthy, comp. cirrhosis, early ASH, non-severe AH, and severe AH models clustered together. Additionally, the NASH and HCV models clustered together. The complete workflow from the Human1 model integrated with RNA-seq data to disease-state-specific GEM generation to structural model comparisons is shown in Figure 2A.

To further compare the GEM structures, we rank ordered the number of reactions, metabolites, genes, and unique gene rules across the models. Those with highest rank (i.e., 1) had the largest number of reactions, metabolites, genes, or unique gene rules, while those with the lowest rank (i.e., 8) had the fewest number of reactions, metabolites, genes, or unique gene rules. We found a consistent pattern, with the AH models having the highest rank, the NALD states the lowest rank, and the healthy state ranked in the middle (Supplementary Figure S1).

Based on the reaction coverage calculated in Supplementary Figure S1, the number of reactions were collated for each biological subsystem and subsequently calculated for each GEM. The number of reactions between any two models differing by more than 10% were determined and the top 10 subsystems with the greatest differences were shown in Figure 2B. Four out of the ten subsystems in Figure 2B were related to sphingolipid metabolism (i.e., sphingolipid metabolism, glycosphingolipid biosynthesis-lacto and neolacto series, glycosphingolipid biosynthesis-ganglio series, and glycosphingolipid metabolism). Previous studies, including one of NAFLD genome-scale metabolic modeling, identify sphingolipid metabolism dysregulation associated with NAFLD progression [25,45]. Therefore, we sought to further analyze the genes responsible for this disparity in reaction coverage across the models. To do so, we found the associated gene rules for each reaction from the sphingolipid subsystems that differed across models. There was a total of 64 reactions, 61 of which had associated gene rules, resulting in 14 unique gene rules. The z-scored expression for each of these genes and the number of reactions included in each model based on the associated gene rule is shown in Figure 2C. A detailed workflow of this analysis beginning with the reaction coverage plot in Figure 2B and ending with a heatmap of gene expression for the sphingolipid-related metabolic genes in Figure 2C is shown in Supplementary Figure S2. The sphingolipid-related gene rules that significantly differentiate the explant AH model from the rest of the models are *Abo*, *St8Sia5*, and *Neu2*. The *Abo* gene encodes proteins related to the blood group system; the *St8Sia5* gene is involved in the synthesis of gangliosides; and the *Neu2* gene encodes a glycohydrolytic enzyme, which removes sialic acid residues from glycoproteins and glycolipids.

### 3.3. Metabolic Functional Analysis of Patients with AH

**Figure 3:**
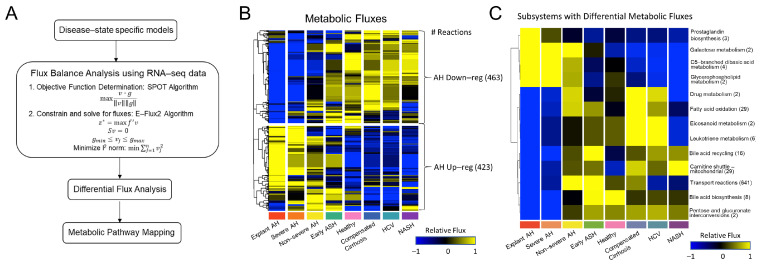
Predicted metabolic flux analysis for the generated GEMs. (**A**). Workflow describing flux balance analysis (FBA) calculations and downstream analysis. (**B**) Hierarchical clustering of metabolic fluxes identified two clusters: (1) AH Down-reg with 463 fluxes and (2) AH Up-reg with 423 fluxes. (**C**) Fluxes were averaged across subsystem and those differing significantly across models, as tested by Wilcoxon ranked sum test (*p* < 0.05), are shown.

To further understand how the structure of these GEMs relates to the function of a diseased and healthy liver, we performed FBA using the *SPOT* and *E-Flux2* algorithm Equations (1)–(5), which are discussed more thoroughly in the methods section. Following FBA, we performed Wilcoxon ranked sum statistical tests to elucidate significant flux differences between the models. The pipeline for FBA and downstream analysis is shown in Figure 3A.

A total of 886 measurable fluxes—which we defined as being greater than 0.1 mmol/gDW/h—were analyzed, plotted in the form of a heatmap, and hierarchically clustered (Figure S3B). Two clusters were identified: one with 423 fluxes that were up-regulated with AH (AH Up-reg) and another with 463 fluxes that were down-regulated with AH (AH Down-reg). The number of reactions within a specific subsystem was summed for each cluster in Figure S3B and tabulated in Supplementary File S12. The subsystems were ranked based on the absolute value of the difference between the number of reactions in each cluster. The AH Down-reg cluster contained all reactions within the fatty acid oxidation pathway (*n* = 29), while the AH Up-reg cluster contained all reactions within the pentose phosphate pathway (*n* = 7).

Fluxes were summed across subsystems and Wilcoxon-ranked sum statistical tests were performed to determine which subsystems had differential flux activity across disease states. The fluxes for each significant subsystem were averaged and visualized in the form of a heatmap (Figure 3C). Hierarchical clustering revealed distinct up- and down-regulated subsystems in AH. However, the non-severe AH fluxes were up-regulated across all subsystems, thereby bridging the gap between the two clusters. Fewer subsystems had up-regulated flux activity with AH; these subsystems included prostaglandin biosynthesis, galactose metabolism, C5-branched dibasic acid metabolism, and glycerophospholipid metabolism. Despite sphingolipid-related subsystems showing significant differences at the GEM structural level in Figure 2, there existed fewer differences at the functional level, as only one of the sphingolipid-related subsystems—glycerophospholipid metabolism, with only two reactions—had significantly higher measurable fluxes in AH. Additionally, despite all pentose phosphate pathway fluxes belonging to the AH Up-reg cluster (Figure 3B and Supplementary File S2), there were no significant flux differences within this pathway across disease states. Instead, most subsystems had significantly lower flux activity in AH, despite what was seen at the structural level, where the AH states had higher reaction, metabolite, and gene coverage (Figure 2B and Supplementary Figure S1). Here, the fatty acid oxidation pathway, whose flux activity solely belongs to the AH Down-reg cluster (Figure 3B and Supplementary File S12), showed significance in Figure 3C. Other subsystems with significantly lower flux activity with AH include leukotriene metabolism, bile acid recycling and biosynthesis, the mitochondrial carnitine shuttle, and transport reactions.

### 3.4. Systems-Level Analysis of Metabolic Regulatory and Functional Variability

**Figure 4:**
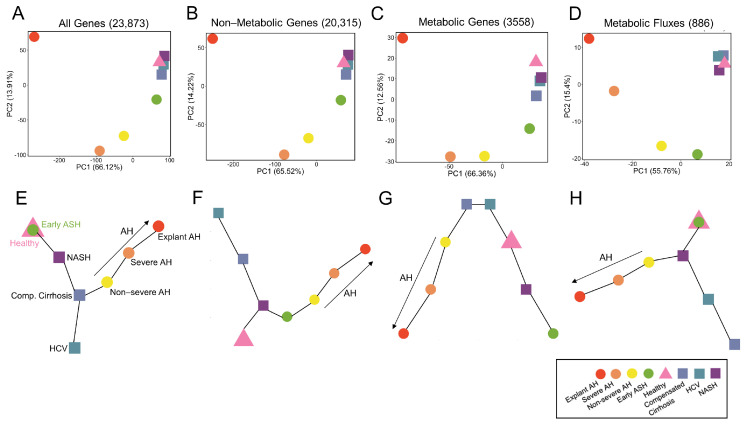
Systems-level analysis of RNA-seq data and predicted metabolic fluxes. Principal component analysis (PCA) plots are shown for (**A**) all 23,873 genes, (**B**) 20,315 non-metabolic genes, (**C**) 3558 metabolic genes, and (**D**) 886 metabolic fluxes. The percent variability for PC1 and PC2 are shown. Minimum spanning trees (MST) are shown for (**E**) all genes, (**F**) non-metabolic genes, (**G**) metabolic genes, and (**H**) metabolic fluxes. In all MST plots, progressing AH disease severity is shown.

We performed a systems-level analysis to determine how the variability across disease states differed at the regulatory (gene expression) vs. functional (predicted metabolic fluxes) level. We performed PCA to analyze such variability for all genes (Figure 4A), non-metabolic genes (Figure 4B), metabolic genes alone (Figure 4C), and predicted metabolic fluxes (Figure 4D). Interestingly, at the gene level (Figure 4A–C), the disease states follow a very similar pattern: the healthy and NALD states cluster closely together, while the early ASH, non-severe AH, and severe AH show a roughly linear progression along PC1 and PC2 as disease severity increases. In all cases, the explant AH state is decently removed from the rest of the AH states, indicating the most disparate gene expression. Still, the explant AH state varies in the direction of the AH states as opposed to clustering with the healthy and NALD states. This result was an intriguing result as the variability of the entire gene space could be explained by (1) including only non-metabolic genes and (2) including only metabolic genes, despite the non-metabolic gene space containing 20,315 genes and the metabolic only gene space containing 3558 genes.

Next, to analyze the variability at the functional level, we generated a PCA plot of the predicted metabolic fluxes for each disease state (Figure 4D). Since the fluxes are calculated by utilizing only metabolic genes, we were interested in comparing the PCA plot of metabolic genes alone (Figure 4C) to the PCA plot of metabolic fluxes (Figure 4D). Similar to the PCA plot for gene expression (Figure 4A–C), the PCA plot for predicted metabolic fluxes (Figure 4D) shows clustering of the healthy and NALD states. However, in the metabolic flux PCA plot all AH samples follow a linear progression in the PC1 and PC2 space from early ASH to explant AH. Therefore, despite utilizing the metabolic gene expression data to calculate the metabolic fluxes, there is an additional layer of complexity that differentiates the functional behavior from the regulatory behavior of the liver for each disease state.

Furthermore, we performed trajectory analyses using MST to determine the pseudo-temporal progression of disease states. Again, this was performed for all genes (Figure 4E), non-metabolic genes (Figure 4F), metabolic genes alone (Figure 4G), and predicted metabolic fluxes (Figure 4H). Despite the PCA plots exhibiting very similar patterns for gene expression (Figure 4A–C), the MSTs show significant differences. The MST for all genes in Figure 4E displays an overlap between the healthy and early ASH states, which progresses towards NASH and then comp. cirrhosis, at which there is a branching point. Along one trajectory is non-severe AH, severe AH, and then explant AH (AH progression), while the other trajectory terminates at HCV. The non-metabolic gene MST in Figure 4F differs in that the healthy state progresses toward the NASH state, which is the branching point. The NASH state diverges towards comp. cirrhosis and then HCV along one trajectory and AH progression along the other trajectory (early ASH, non-severe AH, severe AH, and then explant AH). The MST for metabolic genes alone (Figure 4G) differed from the other regulatory MSTs in that there are two start points, namely comp. cirrhosis and HCV. The comp. cirrhosis branch trends towards progressing AH severity, while the HCV branch trends towards healthy, NASH, and then early ASH state.

The MSTs provide novel information that is not apparent in the PCA plots. While the PCA plot of metabolic genes alone (Figure 4C) shows a similar pattern to the non-metabolic and all gene PCA plots (Figure 4A,B), the MST plot differs as the progression from healthy to AH states is not present. However, when generating an MST for predicted metabolic fluxes (Figure 4H), this progression is recovered and more closely mimics the MSTs for all genes as well as non-metabolic genes (Figure 4E,F), as there is a distinct trajectory from healthy to AH progression. The MSTs highlight the distinct system’s level differences of the regulatory and functional behavior of the healthy and diseased liver.

### 3.5. Glutathione Metabolism Dysregulated in Patients with AH

**Figure 5:**
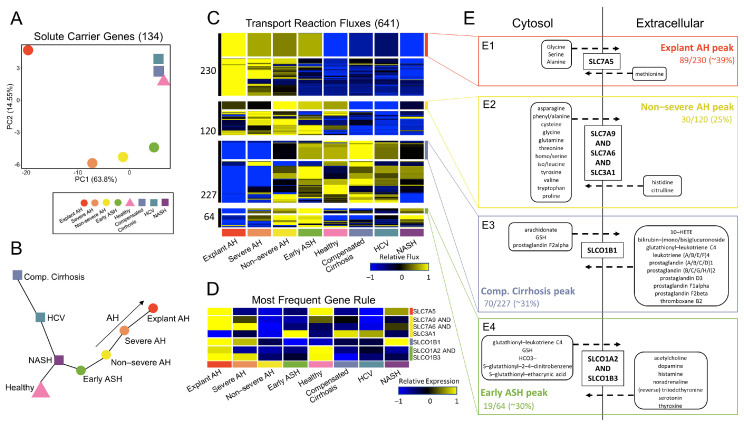
Regulatory and functional activity of solute transporters. (**A**) PCA plot for the 134 solute carrier (SLC) genes. (**B**) MST plot for the SLC genes. (**C**) Hierarchical clustering of fluxes from the transport reaction subsystem reveals four clusters. For each cluster, the gene rule associated with each reaction was identified. The reactions with the most frequent gene rule are annotated for each cluster. (**D**) The associated gene expression for each of the most frequent gene rules in (**C**) is shown. (**E**) A metabolic pathway map for each of the clusters with associated most frequent gene rule is shown. (**E1**) shows sequentially increasing flux behavior with AH severity, peaking at explant AH, for reactions with gene rule SLC7A5. (**E2**) shows increasing flux activity from healthy to non-severe AH, at which the fluxes peak, for reactions with gene rule SLC7A9 and SLC7A6 and SLC3A1. (**E3**) shows fluxes peaking at compensated cirrhosis for reactions with gene rule SLCO1B1. (**E4**) shows sequentially increasing flux behavior with decreasing AH severity, peaking at early ASH, for reactions with gene rule SLCO1A2 and SLCO1B3.

We sought to further investigate the transport reactions, as they constituted the majority of fluxes with differential activity (641 of 746 significant fluxes within the transport reaction subsystem; Figure 3C). We were interested in how the disease states clustered and progressed when PCA and subsequent trajectory analysis were performed. We limited the gene space to include only solute carrier (SLC) genes present in the gene rules of the reactions resulting in significant flux activity; 134 SLC genes fell into this category. These genes are also considered “metabolic” as they are present in all GEMs. Interestingly, the PCA plot, limited to only the 134 SLC genes (Figure 5A), exhibited vast similarity in the progression of AH samples to PCA plots in Figure 4A,B for all genes and non-metabolic genes alone. This is despite these genes belonging to the metabolic genes PCA plot in Figure 4C. Additionally, the MST for SLC genes alone (Figure 5B) was identical to the MST of non-metabolic genes (Figure 4F) for the progression of the healthy state to early ASH state to the AH states of increasing severity.

The PCA plot and MST were able to accurately capture the behavior of the non-metabolic genes while utilizing only the SLC genes, which represent a subset of the metabolic gene space. Therefore, we were interested in further analyzing how the behavior of the 641 fluxes from the transport reaction subsystem led to functional metabolic differences across disease states. Hierarchical clustering of the 641 transport fluxes in Figure 5C elucidated four distinct clusters. Within each of the four clusters, the reactions and corresponding gene rules for each reaction were identified and a frequency table of the gene rules was generated. The reactions, gene rules, metabolic equations, and frequency tables for each cluster can be found in Supplemental File S7. The gene rule with the highest frequency for each cluster was determined and the reactions with these gene rules were highlighted within each of the four clusters (Figure 5C). The associated gene expression was plotted for each of the most frequent solute carrier gene rules to highlight the differences between predicted metabolic activity and regulatory behavior (Figure 5D). A metabolic map of the transported molecules from the four most frequent gene rules are shown in Figure 5E. The metabolic map shows substrates from the transport reactions only if they were involved in export or import but not both.

In Cluster 1, 89 of the 230 transport reaction fluxes (39% of the fluxes) had gene rule *Slc7a5*. The gene *Slc7a5* encodes a L-type amino acid transporter, LAT1, that mediates the influx of neutral essential amino acids (i.e., leucine, isoleucine, phenylalanine, methionine, histidine, tryptophan, valine, and tyrosine) in exchange for the efflux of intracellular substrates, such as other essential amino acids or glutamine [46]. The flux through reactions with this gene rule exhibit a sequential increase in flux with AH severity, peaking at explant AH. In all reactions, methionine is being transported into the cytosol in exchange for glycine, serine, and alanine (Figure 5(E1)).

Cluster 2 has 30 out of the 120 fluxes (25%) with most frequent gene rule *Slc7a9 AND Slc7a6 AND Slc3a1*. The gene rule encodes BAT1, which is involved in the transport of cysteine, arginine, leucine, and glutamine. Within this cluster, there is sequentially increasing flux activity from the healthy state to early ASH to non-severe AH, at which point the flux activity decreases. The reactions with this gene rule export cysteine, glutamine, and leucine (Figure 5(E2)).

Cluster 3 has 70 out of the 227 fluxes (31%) with gene rule *Slco1b1*, which encodes a liver-specific transmembrane receptor, OATP1B1, that mediates the uptake of numerous endogenous compounds, including prostaglandins and leukotriene C4. In this cluster, fluxes increase from AH to early ASH to the healthy state to comp. cirrhosis. Fluxes also increase to a peak at comp. cirrhosis from the NALD side of the disease spectrum (i.e., from NASH to HCV to comp. cirrhosis). The reactions with this gene rule import all prostaglandins, except prostaglandin F2alpha, and leukotriene C4 (Figure 5(E3)). Cluster 4 has 19 out of the 64 fluxes (30%) with gene rule *Slco1a2 AND Slco1b3*, which encodes the enzyme OATP1A2, which is responsible for the uptake of bile acids and other organic anions. These fluxes show an increase in activity with decreasing AH severity, peaking at early ASH. Reactions with this gene rule shuttle out glutathione (GSH) and other glutathione tagged molecules (Figure 5(E4)).

Within the comp. cirrhosis peak cluster, there is influx of prostaglandins (PGs), which are lipids that sustain homeostatic functions and mediate pathogenic mechanisms, including the inflammatory response [47]. PGs have also been shown to play a role in potentiation of the liver regeneration cascade [48]. Therefore, this cluster of fluxes highlights the compensatory behavior of the liver, which is lost with AH.

**Figure 6:**
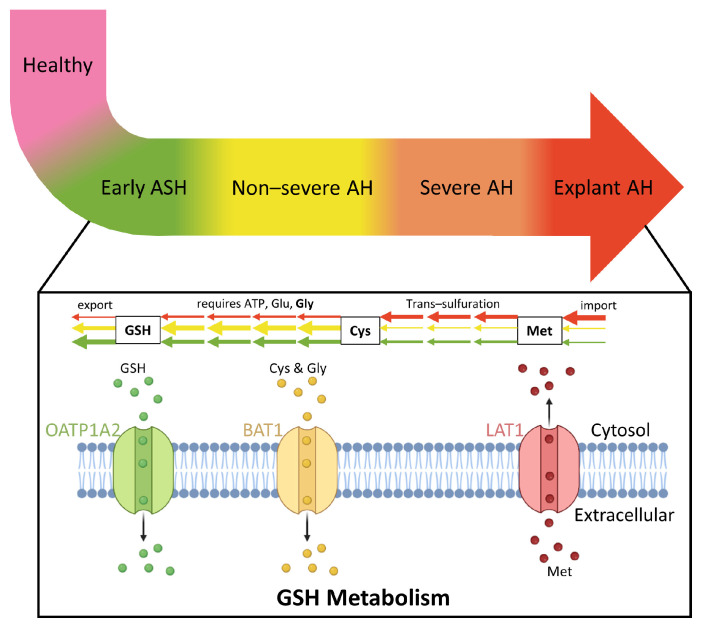
Glutathione (GSH) metabolism and transport activity is sequentially dysregulated with increasing AH severity. As AH severity increases, the earlier the steps in the GSH metabolism pathway are affected. While the early ASH disease state affects the last step in the pathway when GSH is exported, the explant AH state affects the first step in the pathway when methionine is imported to produce cysteine. Created with BioRender.com (accessed on 19 November 2022).

All ALD-related clusters (explant AH peak, non-severe AH peak, and early ASH peak) involve components of GSH metabolism. GSH defends against oxidative stress, participates in detoxification of xenobiotics, determines the redox status of the cell, and regulates vital processes, such as growth and apoptosis [49]. Alteration of this pathway has been directly implicated in liver disease as discussed in a recent review paper [49]. The explant AH peak involves influx of methionine; methionine is converted to cysteine by trans-sulfuration in the initial stage of GSH metabolism. GSH is then synthesized via two ATP-requiring enzymatic steps. The first is the rate limiting step, which requires glutamate, while the second step requires glycine. In the final step of this pathway, GSH is exported into sinusoidal blood and bile. Cysteine and glycine are both exported in the non-severe AH peak cluster, while GSH is exported in the early ASH peak. A detailed schematic of increasing AH severity in coordination with the sequential dysregulation of GSH metabolism is shown in Figure 6.

We sought to validate the predicted metabolic flux data by mining previously published metabolomics studies from patients with AH. One study analyzed serum metabolomics data from patients with severe AH and AC [50]. They found that the abundance of small molecules related to glutathione metabolism were significantly altered. Specifically, they identified significant up-regulation of intermediates alpha-ketobutyrate, 2-hydroxybutyrate, and methionine in individuals with severe AH as compared to individuals with AC. Additionally, there was a trend towards higher levels of 5-oxoproline (*p* = 0.066) in severe AH patients as compared to AC patients. The observed dysregulated glutathione pathway intermediates in the patient serum are consistent with the model-predicted dysregulation of metabolic fluxes in the glutathione metabolic pathway in AH.

## 4. Discussion

The combinatorial analysis of transcriptomic data and predicted metabolic fluxes shown herein provides unique insights into the regulatory and functional behavior of livers from patients with AH. While previous studies have identified dysregulated metabolic flux activity in livers of patients with NALD [24,25], this is the first study of its kind to identify significant alterations in metabolic flux activity associated with AH progression. In this study, we highlight the need for distinct analysis of AH and NALD states as their gene expression, model structure, and metabolic flux behavior differ significantly.

At the regulatory level, genes up-regulated with AH (AH Up-reg) are enriched in specific biological pathways, namely, nucleotide metabolism, PtdIns metabolism, protein ubiquitination, and mRNA transport, while genes down-regulated with AH (AH Down-reg) are enriched in the oxidative phosphorylation pathway. At the model structural level, the regulatory behavior is bridged with the functional behavior of the liver. Reactions, metabolites, and genes are included in the generated GEM if the calculated gene rule, which utilizes the metabolic gene expression, reaches a specified threshold of 1. Here, we found that the reaction, metabolite, and gene coverage was highest in the AH models and increased with disease severity (i.e., non-severe to explant AH). Sphingolipid-related subsystems accounted for the majority of differences in reaction coverage across models.

At the functional level, metabolic fluxes were predicted by FBA, identifying four subsystems with fluxes up-regulated in the livers of patients with AH as compared to the nine subsystems with fluxes down-regulated in AH. Of those subsystems down-regulated with AH, fatty acid oxidation, bile acid metabolism, the mitochondrial carnitine shuttle, and leukotriene metabolism had greater than six reactions present in the subsystem. Additionally, the transport reactions subsystem made up the majority of fluxes with significant differences as roughly 86% of all significant fluxes belong to this subsystem.

PCA plots for all genes, non-metabolic genes, metabolic genes alone, and predicted metabolic fluxes identified similar patterns for AH progression. Interestingly, the PCA plot of predicted fluxes showed a more distinct progression of AH states beginning at early ASH, progressing to non-severe AH, severe AH, and then explant AH. This progression was not accurately captured by any of the PCA plots of gene expression data. Additionally, the MST plot of predicted metabolic fluxes showed a similar pseudo-temporal progression to that of the entire gene space, but to a lesser extent the metabolic only gene space. Therefore, we concluded that the predicted flux values were able to accurately capture the pattern and progression of AH despite only utilizing the metabolic gene expression data. Furthermore, it confirms that the *tINIT* algorithm, which integrates the gene expression data with the Human1 metabolic model through the use of metabolic gene rules and subsequent FBA, was able to identify unique functional capabilities of a diseased liver that are missing when only analyzing the gene expression alone.

Furthermore, we sought to analyze the flux activity from the 641 significant transport reactions. To do so, we first generated a PCA plot and MST for the SLC genes from the gene rules for each of the 641 reactions. The PCA plot of 134 SLC genes was nearly identical to the PCA plots of all genes and non-metabolic genes. Furthermore, the MST of SLC genes showed identical progression of AH samples as seen in the MST of non-metabolic genes. Therefore, we identified a specific gene set (the SLC genes) within the metabolic gene space that had similar behavior to the non-metabolic genes and could thereby properly explain the progression of AH state severity. Hierarchical clustering of the transport reaction fluxes alone identified four distinct clusters associated with progressing AH. The gene rules for each cluster were tabulated and those with the highest frequencies were identified. We then plotted the substrates for each of the most frequent reactions’ gene rule in the form of a metabolic map to highlight the dysregulated reaction activity within each of the four clusters. We identified metabolites in the clusters peaking with alcoholic liver disease (i.e., explant AH peak, non-severe AH peak, early ASH peak) to be involved in GSH metabolism. The metabolites in the comp. cirrhosis peaking cluster were implicated in inflammatory response, highlighting the compensatory mechanism of the liver to respond to non-AH related diseases. Metabolic engineering of the enzymes encoding the most frequent gene rule in each of these transport reaction flux clusters may provide therapeutic potential in mimicking the healthy state. For instance, the enzyme encoding gene rule *Slc7a5*, LAT1, which is most frequent in the explant AH peak cluster, may be utilized therapeutically to aid patients with early ASH and AH but not NALD, as the fluxes in this cluster are down-regulated for healthy and NALD states. Therefore, metabolic engineering of LAT1 in patients with AH such that its flux activity is down-regulated may provide therapeutic relief.

Previous studies have described age-dependent changes in regulation of metabolic pathways [51,52,53]. In the present study, the AH patients were of similar age as early ASH, NASH, and HCV patients, but were older than the healthy controls and younger than patients diagnosed with compensated cirrhosis. The present flux balance modeling and analysis predicted a progressive shift in metabolic dysregulation across the disease states from healthy controls to severe AH. However, the progression of disease stage or severity was not aligned with increasing median age in these patient groups. Considering this lack of alignment, our model predicted progressive dysregulation of metabolic fluxes in ALD may be minimally affected by age-dependent changes.

One consideration for patients with AH is determining the metabolic effects for individuals with co-morbidities such as type 2 diabetes mellitus (T2DM). A previous study showed that individuals with T2DM have glutathione deficiency [54]. While the T2DM status of each individual was not explicitly tracked in the relatively small cohort of AH patients in our study, patients with AH typically do not have a higher prevalence of T2DM in comparison to the rest of the population [55,56]. As of 2022, approximately 11% of the general population have been diagnosed with diabetes in the US, and a previous study on AH revealed that approximately the same percent of individuals diagnosed with AH had T2DM [55,56]. Further investigation is needed with a larger patient cohort in which there is explicit tracking of co-morbidities such as T2DM and metabolic syndrome. Such a study can uncover potential contributions of chronic metabolic diseases to the observed glutathione dysregulation in AH.

The methodology and analysis discussed in this manuscript from liver sample acquisition to flux balance analysis can be fast tracked in the clinic to distinguish between various AH states, thereby allowing the hepatologist to determine which therapeutic avenue is most beneficial. Specifically, if individual patient samples are obtained, sequenced, and then integrated with the Human1 metabolic model, predicted fluxes (by FBA) can be obtained fairly quickly and the extent to which the diseased liver has progressed can be determined based on how the sample clusters with the rest of the disease states. However, future studies must be conducted to determine how blood serum-based sequencing and integration with a metabolic model correlates with the predictive modeling results of sequencing liver tissue, as to limit the invasiveness of taking biopsy samples. Furthermore, individual, patient-specific models of progressing AH should be generated to identify variability within each disease state in addition to differences across each disease state, which was conducted in the current study. Therefore, therapeutics can be personalized to patients based on the regulatory and functional behavior of their livers.

The combinatorial regulatory and functional analysis discussed in this manuscript provides a unique framework for identification of biomarkers differentiating the various stages of AH progression. The current standard of care treatment for patients with AH can be adjusted and improved through possible use of metabolic modulators, thereby tipping the flux behavior of diseased livers towards a healthy state.

## Data Availability

The raw RNA-sequencing data are deposited in the Database of Genotypes and Phenotypes (dbGAP) of the National Center for Biotechnology Information (United States National Library of Medicine, Bethesda, MD) under accession number phs001807.v1.p1. The filtered and normalized data can be found on GitHub (https://github.com/Daniel-Baugh-Institute/AlcoholicHepatitis_LiverGEMStudy (accessed on 19 November 2022)) or as a supplement to the current manuscript.

## Supplementary Materials

The following supporting information can be downloaded at https://www.mdpi.com/article/10.3390/metabo12121157/s1: Figure S1: Reaction, metabolite, gene, and unique gene rule coverage for each GEM rank ordered from 1 (Max) to 8 (Min); Figure S2: Workflow for plotting the gene expression associated with the gene rules for all reactions within the sphingolipid-related subsystems from Figure 2; File S1: Excel file containing the metabolic task list needed for generating the GEMs; File S2: MATLAB script that generates all GEMs using the *tINIT* algorithm; File S3: MATLAB script that calculates the structural comparisons between all GEMs using the *compareMultipleModels* function; File S4: MATLAB script that calculates the model objective functions and performs flux balance analysis; File S5: Zipped file containing all MATLAB functions and scripts required for running the code in Files S2–S4; File S6: Instruction Manual for generating the GEMs and performing structural comparisons between the models; File S7: Zipped file containing all 8 GEM files in .mat format; File S8: R script for replicating the Figures in the present manuscript; File S9: Zipped file containing all necessary files for Figure replication; File S10: File containing the document describing the self-assessed conformance of our models to the state of the art Ten Simple Rules for Credible Practice in Modeling and Simulation in Healthcare; File S11: Excel file containing a comprehensive list of all biological processes significantly enriched (FDR < 0.05) in the AH Up-reg and Down-reg clusters from Figure 1C; File S12: Excel file containing the number of reactions within each subsystem for each metabolic flux cluster in Figure 3B.

## References

[1] Lucey M.R., Mathurin P., Morgan T.R. (2009). Alcoholic Hepatitis. N. Engl. J. Med..

[2] Bertha M., Choi G., Mellinger J. (2021). Diagnosis and Treatment of Alcohol-Associated Liver Disease: A Patient-Friendly Summary of the 2019 AASLD Guidelines. Clin. Liver Dis..

[3] Singal A.K., Walia I., Singal A., Soloway R.D. (2011). Corticosteroids and Pentoxifylline for the Treatment of Alcoholic Hepatitis: Current Status. World J. Hepatol..

[4] Patel R., Mueller M. (2022). Alcoholic Liver Disease. StatPearls.

[5] Dang K., Hirode G., Singal A.K., Sundaram V., Wong R.J. (2020). Alcoholic Liver Disease Epidemiology in the United States: A Retrospective Analysis of 3 US Databases. Am. J. Gastroenterol..

[6] Cholankeril G., Ahmed A. (2018). Alcoholic Liver Disease Replaces Hepatitis C Virus Infection as the Leading Indication for Liver Transplantation in the United States. Clin. Gastroenterol. Hepatol. Off. Clin. Pract. J. Am. Gastroenterol. Assoc..

[7] Gao B., Bataller R. (2011). Alcoholic Liver Disease: Pathogenesis and New Therapeutic Targets. Gastroenterology.

[8] Toshikuni N., Tsutsumi M., Arisawa T. (2014). Clinical Differences between Alcoholic Liver Disease and Nonalcoholic Fatty Liver Disease. World J. Gastroenterol..

[9] Ikejima K., Kon K., Yamashina S. (2020). Nonalcoholic Fatty Liver Disease and Alcohol-Related Liver Disease: From Clinical Aspects to Pathophysiological Insights. Clin. Mol. Hepatol..

[10] Rasineni K., Penrice D.D., Natarajan S.K., McNiven M.A., McVicker B.L., Kharbanda K.K., Casey C.A., Harris E.N. (2016). Alcoholic vs Non-Alcoholic Fatty Liver in Rats: Distinct Differences in Endocytosis and Vesicle Trafficking despite Similar Pathology. BMC Gastroenterol..

[11] Greuter T., Malhi H., Gores G.J., Shah V.H. (2017). Therapeutic Opportunities for Alcoholic Steatohepatitis and Nonalcoholic Steatohepatitis: Exploiting Similarities and Differences in Pathogenesis. JCI Insight.

[12] Wen C.-S., Ho C.-M. (2018). Alcohol or Not: A Review Comparing Initial Mechanisms, Contributing Factors, and Liver Transplantation Outcomes Between Alcoholic and Nonalcoholic Steatohepatitis. EMJ.

[13] Mitra S., De A., Chowdhury A. (2020). Epidemiology of Non-Alcoholic and Alcoholic Fatty Liver Diseases. Transl. Gastroenterol. Hepatol..

[14] Ceni E., Mello T., Galli A. (2014). Pathogenesis of Alcoholic Liver Disease: Role of Oxidative Metabolism. World J. Gastroenterol..

[15] Robinson K.E., Shah V.H. (2020). Pathogenesis and Pathways: Nonalcoholic Fatty Liver Disease & Alcoholic Liver Disease. Transl. Gastroenterol. Hepatol..

[16] Teschke R. (2018). Alcoholic Liver Disease: Alcohol Metabolism, Cascade of Molecular Mechanisms, Cellular Targets, and Clinical Aspects. Biomedicines.

[17] Massey V., Parrish A., Argemi J., Moreno M., Mello A., García-Rocha M., Altamirano J., Odena G., Dubuquoy L., Louvet A. (2021). Integrated Multiomics Reveals Glucose Use Reprogramming and Identifies a Novel Hexokinase in Alcoholic Hepatitis. Gastroenterology.

[18] Argemi J., Latasa M.U., Atkinson S.R., Blokhin I.O., Massey V., Gue J.P., Cabezas J., Lozano J.J., Van Booven D., Bell A. (2019). Defective HNF4alpha-Dependent Gene Expression as a Driver of Hepatocellular Failure in Alcoholic Hepatitis. Nat. Commun..

[19] Mardinoglu A., Agren R., Kampf C., Asplund A., Uhlen M., Nielsen J. (2014). Genome-Scale Metabolic Modelling of Hepatocytes Reveals Serine Deficiency in Patients with Non-Alcoholic Fatty Liver Disease. Nat. Commun..

[20] Agren R., Mardinoglu A., Asplund A., Kampf C., Uhlen M., Nielsen J. (2014). Identification of Anticancer Drugs for Hepatocellular Carcinoma through Personalized Genome-scale Metabolic Modeling. Mol. Syst. Biol..

[21] Naik A., Rozman D., Belič A. (2014). SteatoNet: The First Integrated Human Metabolic Model with Multi-Layered Regulation to Investigate Liver-Associated Pathologies. PLoS Comput. Biol..

[22] Gille C., Bölling C., Hoppe A., Bulik S., Hoffmann S., Hübner K., Karlstädt A., Ganeshan R., König M., Rother K. (2010). HepatoNet1: A Comprehensive Metabolic Reconstruction of the Human Hepatocyte for the Analysis of Liver Physiology. Mol. Syst. Biol..

[23] Wang Y., Eddy J.A., Price N.D. (2012). Reconstruction of Genome-Scale Metabolic Models for 126 Human Tissues Using MCADRE. BMC Syst. Biol..

[24] Hyötyläinen T., Jerby L., Petäjä E.M., Mattila I., Jäntti S., Auvinen P., Gastaldelli A., Yki-Järvinen H., Ruppin E., Orešič M. (2016). Genome-Scale Study Reveals Reduced Metabolic Adaptability in Patients with Non-Alcoholic Fatty Liver Disease. Nat. Commun..

[25] Sen P., Govaere O., Sinioja T., McGlinchey A., Geng D., Ratziu V., Bugianesi E., Schattenberg J.M., Vidal-Puig A., Allison M. (2022). Quantitative Modeling of Human Liver Reveals Dysregulation of Glycosphingolipid Pathways in Nonalcoholic Fatty Liver Disease. iScience.

[26] Dobin A., Davis C.A., Schlesinger F., Drenkow J., Zaleski C., Jha S., Batut P., Chaisson M., Gingeras T.R. (2013). STAR: Ultrafast Universal RNA-Seq Aligner. Bioinformatics.

[27] Li B., Dewey C.N. (2011). RSEM: Accurate Transcript Quantification from RNA-Seq Data with or without a Reference Genome. BMC Bioinform..

[28] Robinson J.L., Kocabaş P., Wang H., Cholley P.-E., Cook D., Nilsson A., Anton M., Ferreira R., Domenzain I., Billa V. (2020). An Atlas of Human Metabolism. Sci. Signal..

[29] Agren R., Liu L., Shoaie S., Vongsangnak W., Nookaew I., Nielsen J. (2013). The RAVEN Toolbox and Its Use for Generating a Genome-Scale Metabolic Model for Penicillium Chrysogenum. PLoS Comput. Biol..

[30] Nilsson A., Nielsen J. (2017). Genome Scale Metabolic Modeling of Cancer. Metab. Eng..

[31] Kim M.K., Lane A., Kelley J.J., Lun D.S. (2016). E-Flux2 and SPOT: Validated Methods for Inferring Intracellular Metabolic Flux Distributions from Transcriptomic Data. PLoS ONE.

[32] Sherman B.T., Hao M., Qiu J., Jiao X., Baseler M.W., Lane H.C., Imamichi T., Chang W. (2022). DAVID: A Web Server for Functional Enrichment Analysis and Functional Annotation of Gene Lists (2021 Update). Nucleic Acids Res..

[33] Thomas P.D., Campbell M.J., Kejariwal A., Mi H., Karlak B., Daverman R., Diemer K., Muruganujan A., Narechania A. (2003). PANTHER: A Library of Protein Families and Subfamilies Indexed by Function. Genome Res..

[34] Szklarczyk D., Gable A.L., Nastou K.C., Lyon D., Kirsch R., Pyysalo S., Doncheva N.T., Legeay M., Fang T., Bork P. (2021). The STRING Database in 2021: Customizable Protein–Protein Networks, and Functional Characterization of User-Uploaded Gene/Measurement Sets. Nucleic Acids Res..

[35] Doncheva N.T., Morris J.H., Gorodkin J., Jensen L.J. (2019). Cytoscape StringApp: Network Analysis and Visualization of Proteomics Data. J. Proteome Res..

[36] Shannon P., Markiel A., Ozier O., Baliga N.S., Wang J.T., Ramage D., Amin N., Schwikowski B., Ideker T. (2003). Cytoscape: A Software Environment for Integrated Models of Biomolecular Interaction Networks. Genome Res..

[37] Bader G.D., Hogue C.W. (2003). An Automated Method for Finding Molecular Complexes in Large Protein Interaction Networks. BMC Bioinform..

[38] Erdemir A., Mulugeta L., Ku J.P., Drach A., Horner M., Morrison T.M., Peng G.C.Y., Vadigepalli R., Lytton W.W., Myers J.G. (2020). Credible Practice of Modeling and Simulation in Healthcare: Ten Rules from a Multidisciplinary Perspective. J. Transl. Med..

[39] García-Ruiz C., Kaplowitz N., Fernandez-Checa J.C. (2013). Role of Mitochondria in Alcoholic Liver Disease. Curr. Pathobiol. Rep..

[40] Middleton P., Vergis N. (2021). Mitochondrial Dysfunction and Liver Disease: Role, Relevance, and Potential for Therapeutic Modulation. Ther. Adv. Gastroenterol..

[41] Meikle P.J., Mundra P.A., Wong G., Rahman K., Huynh K., Barlow C.K., Duly A.M.P., Haber P.S., Whitfield J.B., Seth D. (2015). Circulating Lipids Are Associated with Alcoholic Liver Cirrhosis and Represent Potential Biomarkers for Risk Assessment. PLoS ONE.

[42] French S.W., Bardag-Gorce F., Dufour J.-F., Clavien P.-A., Trautwein C., Graf R. (2005). Ubiquitin-Proteasome Pathway in the Pathogenesis of Liver Disease. Signaling Pathways in Liver Diseases.

[43] Donohue T.M. (2002). The Ubiquitin-Proteasome System and Its Role in Ethanol-Induced Disorders. Addict. Biol..

[44] Park J.-S., Ma H., Roh Y.-S. (2021). Ubiquitin Pathways Regulate the Pathogenesis of Chronic Liver Disease. Biochem. Pharmacol..

[45] Régnier M., Polizzi A., Guillou H., Loiseau N. (2019). Sphingolipid Metabolism in Non-Alcoholic Fatty Liver Diseases. Biochimie.

[46] Häfliger P., Charles R.-P. (2019). The L-Type Amino Acid Transporter LAT1—An Emerging Target in Cancer. Int. J. Mol. Sci..

[47] Ricciotti E., FitzGerald G.A. (2011). Prostaglandins and Inflammation. Arterioscler. Thromb. Vasc. Biol..

[48] Schoen Smith J.M., Lautt W.W. (2005). The Role of Prostaglandins in Triggering the Liver Regeneration Cascade. Nitric Oxide.

[49] Lu S.C. (2020). Dysregulation of Glutathione Synthesis in Liver Disease. Liver Res..

[50] Rachakonda V., Gabbert C., Raina A., Bell L.N., Cooper S., Malik S., Behari J. (2014). Serum Metabolomic Profiling in Acute Alcoholic Hepatitis Identifies Multiple Dysregulated Pathways. PLoS ONE.

[51] Cree M.G., Newcomer B.R., Katsanos C.S., Sheffield-Moore M., Chinkes D., Aarsland A., Urban R., Wolfe R.R. (2004). Intramuscular and Liver Triglycerides Are Increased in the Elderly. J. Clin. Endocrinol. Metab..

[52] Basu R., Dalla Man C., Campioni M., Basu A., Klee G., Toffolo G., Cobelli C., Rizza R.A. (2006). Effects of Age and Sex on Postprandial Glucose Metabolism. Diabetes.

[53] Kim I.H., Kisseleva T., Brenner D.A. (2015). Aging and Liver Disease. Curr. Opin. Gastroenterol..

[54] Lutchmansingh F.K., Hsu J.W., Bennett F.I., Badaloo A.V., McFarlane-Anderson N., Gordon-Strachan G.M., Wright-Pascoe R.A., Jahoor F., Boyne M.S. (2018). Glutathione Metabolism in Type 2 Diabetes and Its Relationship with Microvascular Complications and Glycemia. PLoS ONE.

[55] CDC National Diabetes Statistics Report | Diabetes. https://www.cdc.gov/diabetes/data/statistics-report/index.html.

[56] Lischner M.W., Alexander J.F., Galambos J.T. (1971). Natural History of Alcoholic Hepatitis: I. The Acute Disease. Am. J. Dig. Dis..

