# Supplementary material for: Genome-Scale Metabolic Modeling Reveals Sequential Dysregulation of Glutathione Metabolism in Livers from Patients with Alcoholic Hepatitis
Source: Metabolites. 2022 Nov 22;12(12):1157. doi: 10.3390/metabo12121157 (PMC9788589; doi:10.3390/metabo12121157)
Supplement: Supplementary file 1 [file metabolites-12-01157-s001.zip › File S10.pdf]

Self-assessment of conformance to the Ten Simple Rules of Credible Practice in Modeling and Simulation in Healthcare

Genome scale metabolic modeling reveals sequential dysregulation of glutathione metabolism in livers from patients with alcoholic hepatitis

The following self-assessment is based on the rules specified in Erdemir et al. (2020) and the rubric available at: https://www.imagwiki.nibib.nih.gov/content/10-simple-rules-conformance-rubric

Date of self-assessment: October 21, 2022

Model files and documentation: https://github.com/Daniel-Baugh-Institute/AlcoholicHepatitis_LiverGEMStudy

Rule 1: Define context clearly: Develop and document the subject, purpose, and intended use(s) of the model or simulation.

Current Conformance Level: Comprehensive

Model Context: Genome scale metabolic model of alcoholic hepatitis (AH) states including non-severe, severe, and explant AH with healthy and non-alcoholic liver disease controls.
Primary goal of the model/tool/database: The primary objective of the modeling study was to use the disease-state specific genome scale metabolic models (GEMs), which were generated by integrating a generic GEM (Human1) with RNA-seq data for each disease state, to identify points of metabolic dysregulation with progressing AH severity. To do so, we calculated a suitable objective function by employing an algorithm that maximized the correlation between a flux vector and the gene expression data. We then performed flux balance analysis and bounded the flux vector by the associated gene expression data. Lastly, the flux solution space was further confined by minimizing the Euclidean norm of the flux vector. The modeling methodology presented in the manuscript provides a unique framework for analyzing the metabolic functionality of livers from patients with alcoholic hepatitis through use of gene expression data integrated with a genome scale metabolic model to predict flux activity.
Biological Domain of the Model: metabolism
Structures of the Model: Liver
Spatial Scales Included in the Model: genome-scale
Time Scales Included in the Model: N/A (static model)

Rule 2: Use contextually appropriate data: Employ relevant and traceable information in the development or operation of a model or simulation.

Current Conformance Level: Extensive

**Data for building the model**	**Published?**	**Private?**	**How is credibility checked?**	**Current Conformance Level**
in vitro (primary cells cell, lines, etc.)	N/A	N/A	N/A	N/A
ex vivo (excised tissues)	N/A	N/A	N/A	N/A
in vivo pre-clinical (lower-level organism or small animal)	N/A	N/A	N/A	N/A
in vivo pre-clinical (large animal)	N/A	N/A	N/A	N/A
Human subjects/clinical	Yes	No	the source data is confirmed to meet detailed data requirements for consistency and source description	Extensive

**Data for validating the model**	**Published?**	**Private?**	**How is credibility checked?**	**Current Conformance Level**
in vitro (primary cells cell, lines, etc.)	N/A	N/A	N/A	N/A
ex vivo (excised tissues)	N/A	N/A	N/A	N/A
in vivo pre-clinical (lower-level organism or small animal)	N/A	N/A	N/A	N/A
in vivo pre-clinical (large animal)	N/A	N/A	N/A	N/A
Human subjects/clinical	Yes	No	the source data is confirmed to meet detailed data requirements for consistency and source description	Extensive

<u>Rule 3: Evaluate within context:</u> Perform verification, validation, uncertainty quantification, and sensitivity analysis of the model or simulation with respect to the reality of interest and intended use(s) of the model or simulation.

Current Conformance Level: Extensive

	**Who Does It?**	**When does it happen?**	**How is it done?**	**Current Conformance Level**
**Verification**	Developer	During development	Comparison of model output with published data	Extensive
**Validation**	Lab Member	During development	model was used to reproduce simulations and figures	Extensive
**Uncertainty Quantification**	User performs uncertainty quantification	Can be performed every time the model is run for a new scenario	User discretion	Adequate
**Sensitivity Analysis**	User performs sensitivity analysis on influential parameters	Can be performed after every new simulation	User discretion	Adequate

<u>Rule 4: List limitations explicitly:</u> Provide restrictions, constraints, or qualifications for or on the use of the model or simulation for consideration by the users or customers of a model or simulation.

Current Conformance Level: Comprehensive

**Disclaimer statement (explain key limitations)**	**Who needs to know about this disclaimer?**	**How is this disclaimer shared with that audience?**	**Current Conformance Level**
Models are limited by the gene expression data	Users	Stated in the main text	Comprehensive
Predicted fluxes are limited by the methodology described for objective function determination and flux balance analysis	Users	Stated in the main text	Comprehensive

<u>Rule 5: Use version control:</u> Implement a system to trace the time history of modeling and simulation activities including delineation of each contributors' efforts.

Current Conformance Level: Extensive

	**Naming Conventions?**	**Repository?**	**Code Review?**
**individual modeler**	N/A	Github	Yes
**within the lab**	Yes	Yes	Yes
**collaborators**	N/A	Github	Yes

Rule 6: Document appropriately: Maintain up-to-date informative records of all modeling and simulation activities, including simulation code, model mark-up, scope and intended use of modeling and simulation activities, as well as users' and developers' guides.

Current Conformance Level: Extensive

	**Current Conformance Level**
**Code Commented?**	Extensive: comments made in all script files provided in the supplement and on GitHub
**Scope and intended use described?**	Extensive: described in the main text
**User's Guide**	Extensive: described in the main text and supplemental files
**Developer's Guide?**	Adequate: model development described in methods of main text and supplemental files

<u>Rule 7: Disseminate broadly:</u> Share all components of modeling and simulation activities, including simulation software, models, simulation scenarios and results.

Current Conformance Level: Extensive

**Target Audience(s):**	**"Inner Circle"**	**Scientific Community**	**Public**
**Simulations**			Description of simulations stated in the main text and supplemental files.
**Models**			Model files present in supplementary material and on GitHub.
**Software**			MATLAB and RStudio were used. All of these are publicly available either freely or for a fee.
**Results**			Described in main text
**Implication of Results**			Described in main text

<u>Rule 8: Get independent reviews</u>: Have the modeling and simulation activity reviewed by nonpartisan third-party users and developers.

Current Conformance Level: Extensive

**Reviewer(s) name and affiliation**	**Shaina Robbins (Thomas Jefferson University)**
When was the review performed	October 21, 2022
How was review performed and outcomes of the review?	A member of the research group, not involved in the present study and does not conduct research in liver biology, performed the review.<br>Model scripts were cross-checked for consistency.<br>Simulation results and figures were independently reproduced using the files provided on GitHub.

Rule 9: Test competing implementations: Use contrasting modeling and simulation implementation strategies to check the conclusions of different strategies against each other.

Current Conformance Level: Adequate

	**Yes or No (briefly summarize)**
**Were competing implementations tested?**	Competing implementations were tested and compared by the first author of the paper.
**Did this lead to model refinement or improvement?**	Yes, the model was refined and improved whenever inconsistencies arose.

<u>Rule 10: Conform to standards:</u> Adopt and promote generally applicable and discipline specific operating procedures, guidelines, and regulations accepted as best practices.

Current Conformance Level: Adequate

	**Yes or No (briefly summarize)**
**Are there operating procedures, guidelines, or standards for this type of multiscale modeling?**	Yes, as described in the credible practice of modeling and simulation in healthcare: ten rules from a multidisciplinary perspective [1].
**How do your modeling efforts conform?**	Our model is implemented in the widely used MATLAB platform for computational modeling. We also used another freely available and popular software, RStudio. The code is commented at critical locations to aid the reader.

References:

1. Erdemir, A.; Mulugeta, L.; Ku, J.P.; Drach, A.; Horner, M.; Morrison, T.M.; Peng, G.C.Y.; Vadigepalli, R.; Lytton, W.W.; Myers, J.G. Credible Practice of Modeling and Simulation in Healthcare: Ten Rules from a Multidisciplinary Perspective. *J. Transl. Med.* **2020**, *18*, 369, doi:10.1186/s12967-020-02540-4.
