# Supplementary material for: Genome-Scale Metabolic Modeling Reveals Sequential Dysregulation of Glutathione Metabolism in Livers from Patients with Alcoholic Hepatitis
Source: Metabolites. 2022 Nov 22;12(12):1157. doi: 10.3390/metabo12121157 (PMC9788589; doi:10.3390/metabo12121157)
Supplement: Supplementary file 1 [file metabolites-12-01157-s001.zip › File S6.pdf]

Instruction Manual:

"Genome-scale metabolic modeling reveals sequential dysregulation of glutathione metabolism in livers from patients with alcoholic hepatitis"

Components of instructions manual:

(1) Downloading/Installing toolboxes and organizing workspace
(2) Generating genome scale metabolic models (GEMs)
(3) Structural Analysis of GEMs
(4) Calculating objective functions using SPOT and flux balance analysis using E-Flux2
(5) MATLAB, RStudio, and RStudio package versions used

Because of the intense computational power necessary for generating the genome scale metabolic models, we suggest running, at minimum, **Part 2 - Generating genome scale metabolic models** (below) on a server. We utilized a 56-core server with Intel Xeon CPU E5-2697.

Whether you choose to run this code on a computational server or locally, you must have a working version of MATLAB available. The instructions and example code provided herein are specified for individuals choosing to run the code on a computational server. However, the same principles can be applied for individuals choosing to run the code locally.

All supplementary files can be downloaded from GitHub (https://github.com/Daniel-Baugh-Institute/AlcoholicHepatitis_LiverGEMStudy) or from the paper supplement.

Part 1 - Downloading/Installing toolboxes and organizing workspace

1. Create a directory named "GEMGeneration".
   Example code: mkdir 'GEMGeneration'
2. Download Human-GEM model from https://github.com/SysBioChalmers/Human-GEM (ver. 1.6.0). Place this folder in the "GEMGeneration" directory.
3. Download RAVEN toolbox from https://github.com/SysBioChalmers/RAVEN (ver. 2.4.1). Place this toolbox in the "GEMGeneration" directory.
4. Download and install Gurobi optimizer here: https://www.gurobi.com/downloads/gurobi-optimizer-eula/ (ver. 8.1.1). Link the optimizer to your working version of Matlab using the provided instructions from Gurobi.
5. Place the following supplementary file in the "GEMGeneration" directory on the server using a file transfer protocol: "DiseaseData_TPMnorm.txt" (File S9 in paper supplement).
   Example code: sftp 'name of server'
   put 'DiseaseData_TPMnorm.txt' 'GEMGeneration'
6. Place the following supplementary files in the "GEMGeneration" directory on the server using a file transfer protocol: "GenerateDiseaseGEM.m" (File S2 in paper supplement) and "StructuralComparisons.m" (File S3 in paper supplement).
   Example code: sftp 'name of server'
   put 'GenerateDiseaseGEM.m' 'GEMGeneration'

put ‘StructuralComparisons.m’ ‘GEMGeneration’

7. Create a directory within the “GEMGeneration” folder named “DiseaseGEM”.
   Example code: mkdir ‘GEMGeneration/DiseaseGEM’

Part 2 - Generating genome scale metabolic models

1. Since the generation of GEM’s may take a long time, you may want to start a new screen in the server so that the code continues to run even if you disconnect from the server. Learn more about the screen command here: https://linuxize.com/post/how-to-use-linux-screen/
   Example Code: screen
2. Start up Matlab on the server
   Example Code: matlab
3. Add “GEMGeneration” directory to your Matlab path.
   Example Code: addpath(genpath(‘GEMGeneration’))
4. Run ‘GenerateDiseaseGEM.m’ script in Matlab
   Example Code: GenerateDiseaseGEM

Part 3 - Structural Analysis of GEMs

1. Start up Matlab on the server
   Example Code: matlab
2. Add “GEMGeneration” directory to your Matlab path.
   Example Code: addpath(genpath(‘GEMGeneration’))
3. Run “StructuralComparisons.m” script in Matlab
   Example Code: StructuralComparisons
4. Close Matlab and use a file transfer protocol to put the generated GEM files and structural comparison files in your local computer directory
   Example Code: quit(matlab)
   sftp ‘name of server’
   get ‘GEMGeneration/DiseaseGEM’ .

Part 4 – Calculating objective functions using SPOT and flux balance analysis using E-Flux2

1. Create another directory on the same level as the “GEMGeneration” directory called “FluxBalanceAnalysis”
   Example code: mkdir ‘FluxBalanceAnalysis’
2. Place the following supplementary file in the “FluxBalanceAnalysis” directory on the server using a file transfer protocol: “Human-GEM-reactions.txt” (File S5 in paper supplement)
   Example code: sftp ‘name of server’
   put ‘Human-GEM-reactions.txt’ ‘FluxBalanceAnalysis’
3. Place the following supplementary files in the “FluxBalanceAnalysis” directory on the server using a file transfer protocol: “flux_balance.m” (File S4 in paper supplement5, “solve_milp.m” (File S5 in paper supplement), “mat_SPOTformat.m” (File S5 in paper

supplement), “SpotEflux2.m” (File S5 in paper supplement), and “RunSpotEflux2.m” (File S4 in paper supplement).

```
Example code: sftp ‘name of server’
              put ‘flux_balance.m’ ‘FluxBalanceAnalysis’
              put ‘solve_milp.m’ ‘FluxBalanceAnalysis’
              put ‘mat_SPOTformat.m’ ‘FluxBalanceAnalysis’
              put ‘SpotEflux2.m’ ‘FluxBalanceAnalysis’
              put ‘RunSpotEflux2.m’ ‘FluxBalanceAnalysis’
```

4. Start up Matlab on the server and add both “GEMGeneration” and “FluxBalanceAnalysis” directories to your Matlab path

```
Example Code: matlab
              addpath(genpath(‘GEMGeneration’))
              addpath(genpath(‘FluxBalanceAnalysis’))
```

5. Run the SPOT and E-Flux2 algorithms by running the ‘RunSpotEflux2.m’ script to calculate the objective function and perform flux balance analysis, respectively. The script saves the generated objective functions, fluxes and models in ‘.mat’ format. It also saves a matrix of all fluxes and a matrix of all fluxes greater than 0.1 to files in ‘.txt’ format.

```
Example Code: RunSpotEflux2
```

6. Close Matlab and use a file transfer protocol to put the file containing all fluxes in your local computer directory. You may transfer the additional files generated from the RunSpotEflux2 script, however they are not necessary for downstream analysis. All files can be found in the “FluxBalanceAnalysis” directory.

```
Example Code: quit(matlab)
              sftp ‘name of server’
              get ‘FluxBalanceAnalysis/DiseaseStateGEM_allfluxes.txt’ .
```

Note: The “DiseaseStateGEM_allfluxes.txt” file downloaded from the server is the same as the “DiseaseState_Fluxes.txt” file in the ‘FigureReplicationFiles.zip’ folder of the supplement.

Part 5 – MATLAB, RStudio, and package versions used

Software:
MATLAB – R2021a
RStudio – 4.2.1

RStudio packages:
dataVisEasy – 0.3.2
limma – 3.52.4
ica - 1.0-3
matrixStats - 0.62.0
pheatmap - 1.0.12
pcaMethods - 1.88.0

tidyverse - 1.3.2
dplyr - 1.0.10
reshape2 - 1.4.4
stats - 4.1.1
ape – 5.6-2
