# Supplementary material for: Genome-Scale Metabolic Modeling Reveals Sequential Dysregulation of Glutathione Metabolism in Livers from Patients with Alcoholic Hepatitis
Source: Metabolites. 2022 Nov 22;12(12):1157. doi: 10.3390/metabo12121157 (PMC9788589; doi:10.3390/metabo12121157)
Supplement: Supplementary file 1 [file metabolites-12-01157-s001.zip › SupplementaryFigures.pdf]

| Disease State | # Reactions (Rank) | # Metabolites (Rank) | # Genes (Rank) | # Unique Gene Rules (Rank) |
|---|---|---|---|---|
| Explant AH | 8852 (1) | 7041 (1) | 3018 (1) | 1521 (1) |
| Severe AH | 8631 (2) | 6911 (2) | 2785 (2) | 1476 (2) |
| Non–severe AH | 8605 (3) | 6896 (3) | 2726 (3) | 1465 (3) |
| Early ASH | 8581 (4) | 6869 (5) | 2688 (4) | 1455 (4) |
| Healthy | 8566 (5) | 6873 (4) | 2622 (5) | 1449 (5) |
| Comp. Cirrhosis | 8551 (6) | 6864 (6) | 2614 (6) | 1444 (6) |
| HCV | 8387 (7) | 6766 (8) | 2445 (7) | 1417 (7) |
| NASH | 8320 (8) | 6782 (7) | 2420 (8) | 1414 (8) |

Rank 8 7 6 5 4 3 2 1
Min. Max.

**Supplementary Figure S1.** Reaction, metabolite, gene and unique gene rule coverage for each GEM rank ordered from 1 (Max) to 8 (Min).

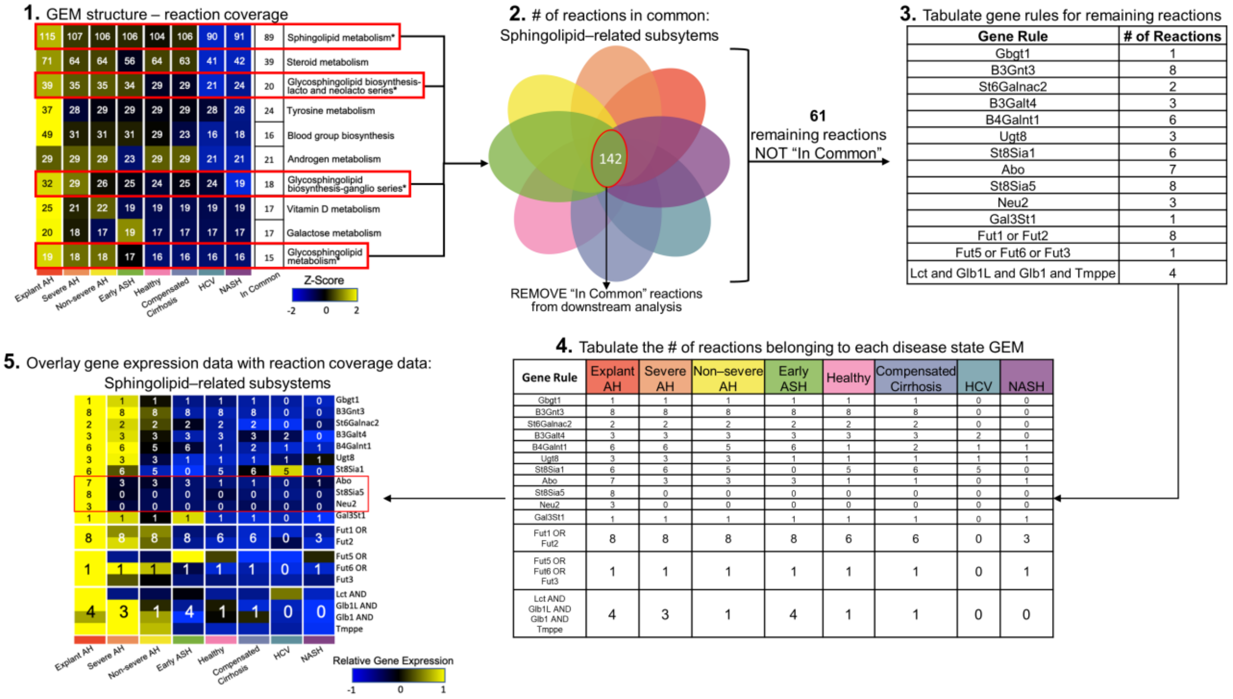


**Supplementary Figure S2.** Workflow for plotting the gene expression associated with the gene rules for all reactions within the sphingolipid-related subsystems from **Figure 2**. (**1**) Reaction coverage for each GEM is plotted as in **Figure 2B**. (**2**) Remove the 142 sphingolipid-related subsystem reactions that are in common across all models. 61 reactions remain and are not in common across the models (**3**) Tabulate the gene rules associated with each reactions and then generate a frequency table of those gene rules. (**4**) For each of the gene rules in the frequency table, determine which of those reactions belong to each GEM. (**5**) Plot the gene expression for each gene within the gene rules and overlay the number of reactions in each of the GEMs with that gene rule as in **Figure 2C**.
